# Concurrent AI-human interaction in prostate cancer MRI interpretation: More hype than help?

**DOI:** 10.1186/s41747-026-00695-1

**Published:** 2026-03-30

**Authors:** Andrea Ponsiglione, Giuseppe Di Costanzo, Alfonso Maria Ponsiglione, Ciro Riccio, Andrea Rinaldo, Anna Giacoma Tucci, Lorenzo Pinto, Luigi Palumbo, Francesca Angelone, Francesco Amato, Arnaldo Stanzione, Renato Cuocolo, Rossano Girometti, Anwar R. Padhani, Massimo Imbriaco

**Affiliations:** 1 Department of Advanced Biomedical Sciences, University of Naples Federico II, Naples, Italy; 2 Department of Radiology, Santa Maria Delle Grazie Hospital, ASL Napoli 2 Nord, Pozzuoli, Italy; 3 Department of Electrical Engineering and Information Technology, University of Naples Federico II, Naples, Italy; 4 Department of Medicine, Surgery and Dentistry, University of Salerno, Baronissi, Italy; 5 Institute of Radiology, Department of Medicine (DMED), University of Udine, University Hospital S. Maria della Misericordia, Azienda Sanitaria Universitaria Friuli Centrale (ASUFC), Udine, Italy; 6 Paul Strickland Scanner Centre, Mount Vernon Cancer Centre, Northwood, UK

**Keywords:** Artificial intelligence, Diagnosis (computer-assisted), Multiparametric magnetic resonance imaging, Prostate-specific antigen, Prostatic neoplasms

## Abstract

**Objective:**

We evaluated a commercial artificial intelligence (AI) system as a concurrent decision-support tool for clinically significant prostate cancer (csPCa) detection.

**Materials and methods:**

In our retrospective study, consecutive patients underwent multiparametric MRI for clinical suspicion of PCa. All scans were reviewed by six readers with varying expertise (two expert radiologists, > 1,000 cases; two basic radiologists, 400‒1,000 cases; and two residents), with and without AI assistance. Intra-/inter-reader agreements and the impact of AI-assistance on patient-level csPCa scores and diagnostic performance, as well as benefit-to-harm ratios, were assessed.

**Results:**

The population consisted of 100 patients with a 26% prevalence of csPCa. There was no improvement in inter-reader agreement with AI-assistance *versus* without (Fleiss κ 0.573 and 0.584, respectively). Residents were most likely to change PI-RADS scores on AI-assisted readings compared to basic and expert radiologists (19, 9, and 7 changes, respectively). Overall, there was no significant difference in area under the receiving operating characteristic curve between AI-assisted and AI-unassisted readings (0.87 *versus* 0.86; *p* = 0.734). At a PI-RADS ≥ 3 threshold, sensitivity was slightly lower with AI (0.87 *versus* 0.89), while specificity (0.73), positive predictive value (0.53–0.54), and negative predictive value (0.94–0.95) remained similar. Subgroup analyses showed no significant differences in diagnostic performance. A slight increase in grade selectivity and selective biopsy avoidance rate was observed among experts and residents, respectively, with AI-assisted readings when applying a PI-RADS cutoff of 3 or PSA density ≥ 0.15 ng/mL/mL.

**Conclusions:**

AI did not significantly improve diagnostic accuracy across readers of varying expertise, with minor impacts on benefit-to-harm ratios.

**Relevance statement:**

We found that AI support in prostate MRI did not significantly improve diagnostic accuracy across readers of varying experience, highlighting the need for further research to optimize AI integration and define its most clinically meaningful roles in prostate cancer detection.

**Key points:**

- Residents were most prone to PI-RADS score modifications after AI-assisted readings compared to AI-unassisted and expert readers.
- There was no significant difference in diagnostic performance metrics between AI-assisted and unassisted readings.
- A slight improvement in grade selectivity among experts and in selective biopsy avoidance among residents was observed during AI-assisted readings for biopsy recommendations.

**Graphical Abstract:**

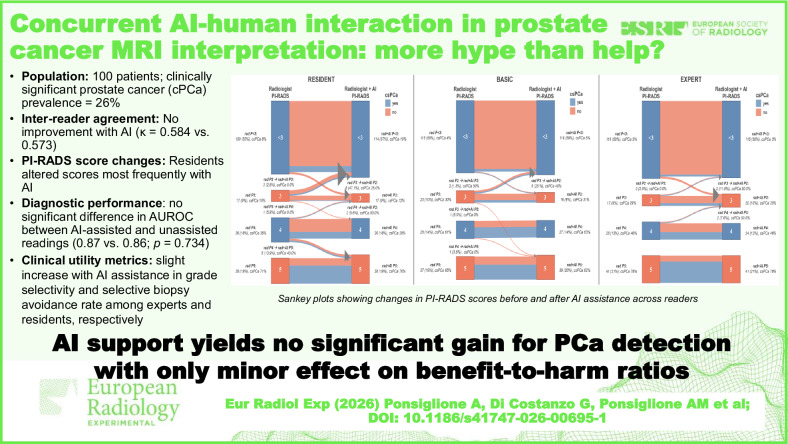

## Background

Performing a prostate MRI before a biopsy has proven ability to decrease unnecessary biopsies, improve the identification of clinically significant prostate cancer (csPCa), and reduce the detection of indolent tumors [1–5]. Despite these benefits, this technique is still hindered by its moderate specificity and high reader variability [6]. Notably, inter-reader variability in Prostate Imaging–Reporting and Data System (PI-RADS) scoring and diagnostic accuracy is influenced by the reader’s experience and the quality of the scans [7–9].

Interest in artificial intelligence (AI) solutions for MRI analysis has surged in recent years, particularly for PCa detection [10]. Most AI tools for prostate MRI include automatic prostate segmentation and volumetry, enabling precise prostate-specific antigen (PSA) density calculation, often surpassing the traditional ellipsoid method [11]. Moreover, AI-driven structured report generation enhances report clarity and consistency [12, 13]. While much of the evidence is retrospective, initiatives like the prostate imaging-cancer AI (PI-CAI) challenge have demonstrated AI’s potential for PCa detection using extensive datasets and diverse readers [14]. However, the available literature on CE-certified AI software for prostate MRI primarily focuses on technical and clinical feasibility, or standalone performance [15, 16]. Therefore, there is a paucity of evidence evaluating AI as a concurrent reader [17]. To address this gap, we aimed to assess the impact of an AI system used as a concurrent decision-support tool for PCa detection involving radiologists with varying levels of expertise in prostate MRI.

## Materials and methods

**Fig. 1:**
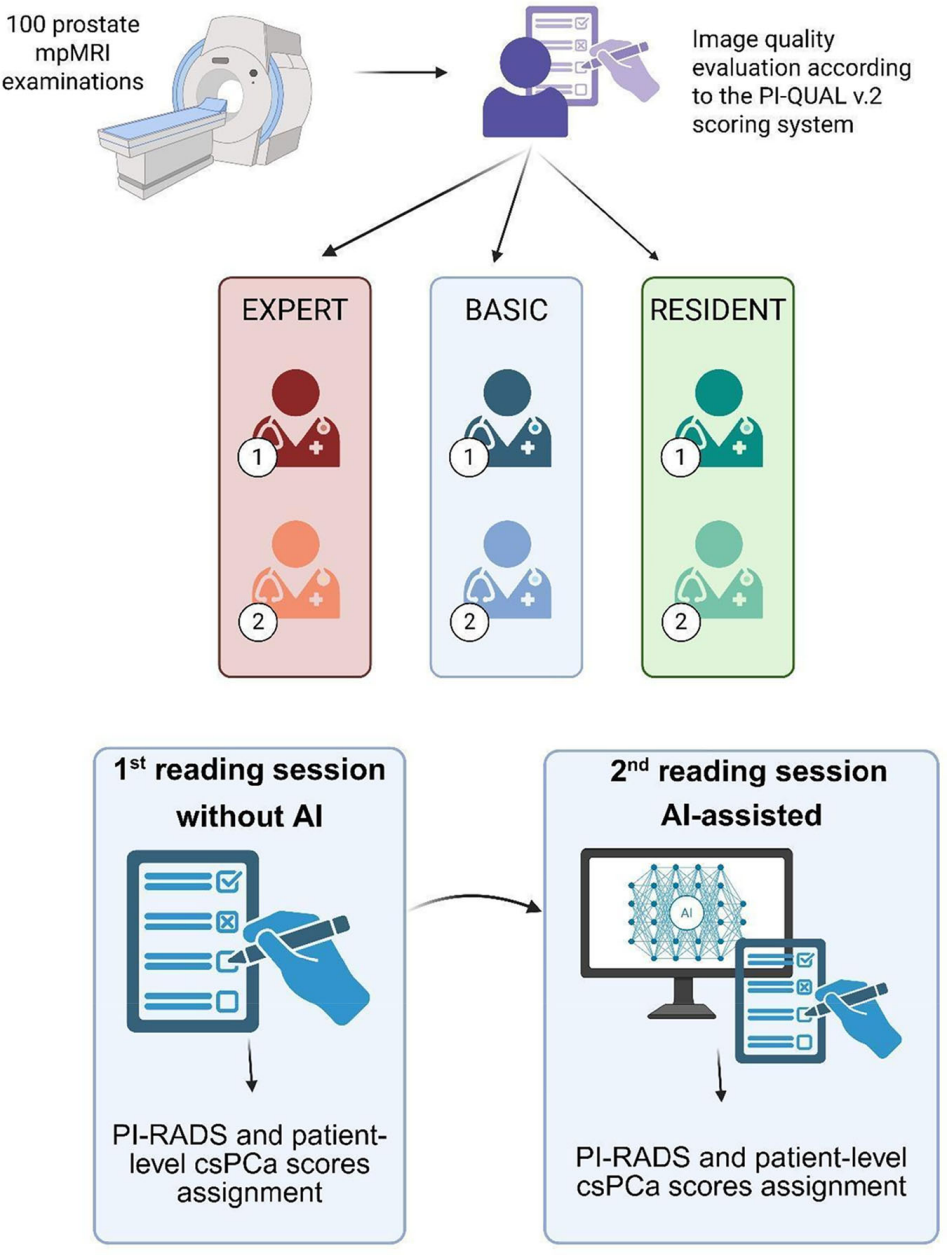
Study outline. The figure was created with BioRender.com's free version

The need for informed consent was waived by the institutional review board, which approved this retrospective study. The study outline is shown in Fig. 1.

### Dataset

We collected scans from 140 consecutive patients undergoing prostate MRI for suspected PCa (PSA ≥ 3 ng/mL and/or positive digital rectal examination) between April 2019 and June 2020. To reduce bias toward MRI-positive cases, MRI-negative patients (PI-RADS 1–2) with follow-up were included, defined as stable/decreasing PSA or stable/resolving MRI at 2 years. Exclusion criteria were: (1) incomplete multiparametric MRI (mpMRI); (2) prior radical PCa treatment; and (3) lack of pathology or follow-up.

### **Demographic and pathological data**

Demographic information, PSA levels, and biopsy results were extracted. Biopsies were performed by two urologists, targeting PI-RADS ≥ 3 lesions (2–4 cores) plus 12 systematic cores. MRI-negative patients with persistently elevated PSA underwent systematic biopsy. Histopathological evaluations were conducted by an experienced genitourinary pathologist (> 15 years of expertise), following the guidelines of the 2014 consensus statement of the International Society of Urological Pathology (ISUP) [18]; csPCa was defined as a lesion with a Gleason score of 3 + 4 or higher.

### MRI protocol

Prostate MRI examinations were conducted using a 1.5-T scanner (MAGNETOM Avanto Fit, Siemens Healthineers, Erlangen, Germany) with a multiparametric approach, adhering to the PI-RADS v2.1 standard [5]. Image acquisition details are given in Supplementary Table S1.

### Image analysis

All mpMRI scans were reviewed by six readers: two experts (> 1,000 cases read), two basic (400‒1,000 cases read), and two residents, per ESUR/ESUI criteria [19]. Each radiologist first interpreted the MRI without the aid of AI, followed immediately by AI-assisted reading (AI-reading). A commercially available advanced imaging and visualization platform designed for PI-RADS reporting, incorporating AI-driven prostate lesion detection and classification (syngo.via MR Prostate AI, version VB50, Siemens Healthineers), was employed. Additional algorithm details, including the pipeline and training, are given in the Supplementary material.

Readers were provided with patient metadata (age, PSA, and PSA density [PSAd])) and asked to assign a PI-RADS v2.1 score to the index lesion, estimate the patient-level likelihood of clinically significant cancer on a 0–100 subjective scale, and document lesion location. The most experienced reader assessed image quality using Prostate Imaging Quality (PI-QUAL) v2 [20].

### Statistical analysis

Continuous variables were reported as medians (interquartile range) and categorical variables as counts (percentages). Inter- and intra-reader agreement for PI-RADS was evaluated with weighted Cohen and Fleiss κ, while likelihood score agreement was assessed with the intraclass correlation coefficient (ICC). The interpretation of the results was as follows: < 0.00, no agreement; 0.01–0.20, slight; 0.21–0.40, fair; 0.41–0.60, moderate; 0.61–0.80, substantial; 0.81–1.00, almost perfect [21]. To evaluate the impact of AI-assisted readings on PI-RADS score and csPCa likelihood suspicion score across different expertise levels, statistical mixed models, including a Cumulative Link Mixed Model‒CLMM and a Generalized Linear Mixed Model‒GLMM for repeated measures, were adopted, considering the reading approach (AI-assisted *versus* AI-unassisted) and the expertise level (resident, basic, or expert, as defined above) as fixed effects. Diagnostic performance for csPCa and any PCa was evaluated using the area under the receiver operating characteristic curve (AUROC) based on patient-level suspicion scores. Sensitivity, specificity, positive predictive value (PPV), and negative predictive value (NPV) were calculated at PI-RADS cutoffs of 3 and 4. Averaged AUROCs across expertise groups were compared with a Wald test, and 95% confidence intervals (CIs) were estimated by bootstrapping.

The rates of Gleason score ≥ 7 cancer detected, biopsies performed, Gleason < 7 cancer detected, and Gleason ≥ 7 cancer missed, with and without AI assistance, were calculated across four biopsy recommendation strategies: (i) radiologist only: PI-RADS ≥ 3 or PSAd ng/ml/mL ≥ 0.15; (ii) radiologist only: PI-RADS ≥ 4 or PSAd ≥ 0.15 ng/mL/mL; (iii) AI-assisted: PI-RADS ≥ 3 or PSAd ≥ 0.15 ng/mL/mL; and (iv) AI-assisted: PI-RADS ≥ 4 or PSAd ≥ 0.15 ng/mL/mL. Grade selectivity, biopsy selectivity, efficiency, and selective avoidance ratios were also estimated according to the following formulas and compared across the above-mentioned recommendation strategies and radiologists’ expertise level [22]:

$${{\rm{Grade}}}\; {{\rm{selectivity}}}=({{\rm{GG}}}\ge 2\,{{\rm{PCa}}})/({{\rm{GG}}}1\,{{\rm{PCa}}})$$

$${{\rm{Biopsy}}}\; {{\rm{efficiency}}}=({{\rm{GG}}}\ge 2\,{{\rm{PCa}}})/({{\rm{GG}}}1\,{{\rm{PCa}}}+{{\rm{benign}}}\; {{\rm{biopsies}}})$$

$${{\rm{Selective}}}\; {{\rm{biopsy}}}\; {{\rm{avoidance}}}=({{\rm{avoided}}}\; {{\rm{biopsies}}})/({{\rm{benign}}}\; {{\rm{biopsies}}})$$

where GG1 stands for grade group 1 (PCa with Gleason score 6).

All statistical analyses have been carried out in R (version 4.4.1) and in Python (version 3.11). A significance level alpha of 0.05 (95% CI) was chosen for all statistical tests.

## Results

### Patient population

Of the 140 eligible patients, we excluded incomplete examinations due to biparametric protocols (*n* = 15), incomplete examinations for claustrophobia (*n* = 3), history of prior radical treatment (*n* = 12), or absence of pathological confirmation or conclusive follow-up data (*n* = 10). Thus, the final study population included 100 patients. Clinical, demographic, and pathological characteristics of the study population are summarized in Table 1. The median patient age was 67 years, with a median PSA level of 8.7 ng/mL. The prevalence of CsPCa was 26% of patients, while any PCa was detected in 38%. Twenty-two patients (median PSA = 6.6 ng/mL) were classified as negative, based on either stable or decreasing PSA values (*n* = 15) or a stable MRI at 2-year follow-up (*n* = 7). Most MRI scans were of acceptable or optimal quality (PI-QUAL 2–3).

**Table 1:** Demographic and clinical characteristics of the patients included in the study

Age, years	67 (62–72)
PSA, ng/mL	8.7 (5.5–12.7)
DRE	
Abnormal	11 (11)
Normal	67 (77)
Not performed	22 (22)
Previous biopsy	
Yes	32 (32)
No	68 (68)
csPCa	
Yes	26 (26)
No	74 (74)
Any PCa	
Yes	38 (38)
No	62 (62)
PI-QUAL score	
Score 2	3 (3)
Score 3	97 (97)
ISUP	
Negative	62 (62)
Grade 1	12 (12)
Grade 2	8 (8)
Grade 3	3 (3)
Grade 4	10 (10)
Grade 5	5 (5)

Continuous data are presented as median and interquartile range. Categorical data are presented as counts and percentages

*PSA* Prostate-specific antigen, *DRE* Digital rectal examination, *csPCa* Clinically significant prostate cancer, *PI-QUAL* Prostate Imaging Quality, *ISUP* International Society of Urological Pathology

### Impact of AI-assisted readings

Table 2 and Supplementary Table S2 report the agreement analysis across the readers with and without the support of AI software. Intra-reader agreement for PI-RADS scores was almost perfect across all radiologists (Cohen κ > 0.950), regardless of AI use. Inter-reader agreement for PI-RADS scores was moderate-to-substantial (Fleiss κ 0.584 without AI *versus* 0.573 with AI). For csPCa likelihood scores, intra-reader agreement was almost perfect (ICC > 0.950), while inter-reader agreement was substantial-to-almost perfect (ICC 0.756 without AI *versus* 0.779 with AI).

**Table 2:** Summary of intra- and inter-reader agreement statistics, reported for overall readings and stratified by radiologist experience level

	Radiologist ID	κ^*^	*p*-value	95% CI	
				Lower limit	Upper limit
Intra-reader agreement (radiologist only *versus* radiologist + AI)					
Expert	1	1.000	< 0.001	1.000	1.000
Expert	2	0.977	< 0.001	0.960	0.995
Basic	3	0.985	< 0.001	0.971	1.000
Basic	4	0.975	< 0.001	0.947	1.000
Resident	5	0.965	< 0.001	0.943	0.988
Resident	6	0.972	< 0.001	0.955	0.990
Inter-reader agreement (radiologist only)					
Experts	1 vs 2	0.895	< 0.001	0.837	0.953
Basics	3 vs 4	0.825	< 0.001	0.731	0.918
Residents	5 vs 6	0.862	< 0.001	0.779	0.945
Overall	All	0.584	< 0.001	0.552	0.617
Overall (PI-RADS = 2)	All	0.680	< 0.001	0.629	0.730
Overall (PI-RADS = 3)	All	0.186	< 0.001	0.135	0.237
Overall (PI-RADS = 4)	All	0.472	< 0.001	0.421	0.523
Overall (PI-RADS = 5)	All	0.735	< 0.001	0.684	0.786
Inter-reader agreement (radiologist + AI)					
Experts	1 vs 2	0.871	< 0.001	0.802	0.940
Basics	3 vs 4	0.811	< 0.001	0.707	0.914
Residents	5 vs 6	0.842	< 0.001	0.748	0.936
Overall	All	0.573	< 0.001	0.540	0.606
Overall (PI-RADS = 2)	All	0.660	< 0.001	0.609	0.710
Overall (PI-RADS = 3)	All	0.133	< 0.001	0.083	0.184
Overall (PI-RADS = 4)	All	0.472	< 0.001	0.421	0.523
Overall (PI-RADS = 5)	All	0.720	< 0.001	0.669	0.770

^*^ Weighted Cohen κ (squared weights) is reported for pairwise comparisons, Fleiss κ for overall comparisons

Although AI assistance did not meaningfully change PI-RADS scores, it significantly increased residents’ overall likelihood scores for csPCa by approximately 9% (Supplementary Table S3 and Supplementary Fig. S1). In contrast, basic and expert radiologists showed only modest, nonsignificant increases of around 3%.

**Fig. 2:**
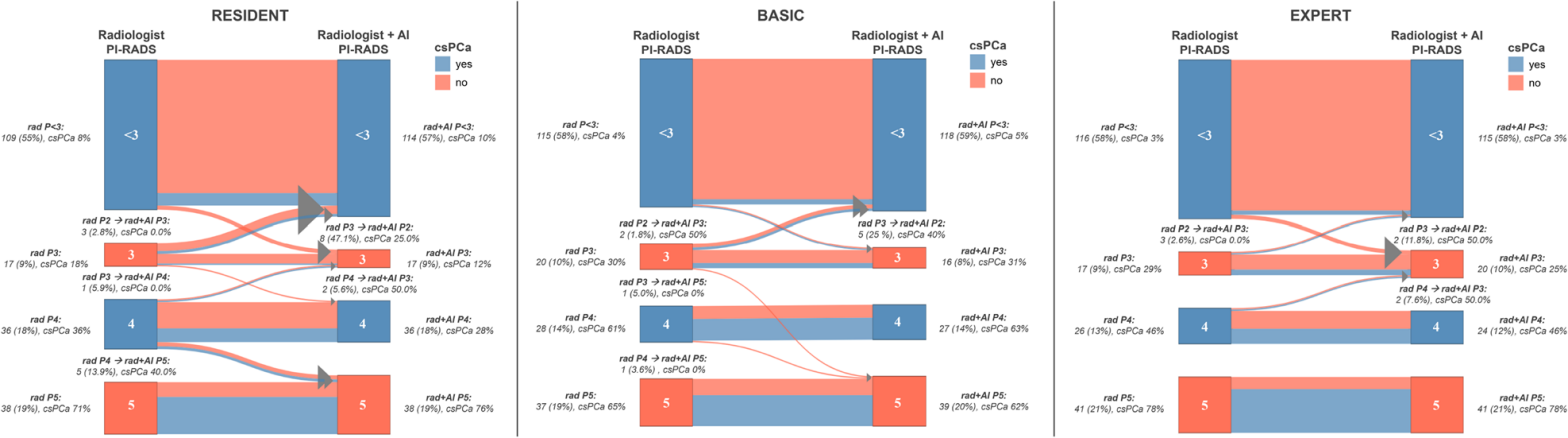
Sankey plots showing changes in PI-RADS scores before (radiologist PI-RADS, rad P) and after (radiologist + AI PI-RADS, rad + AI P) AI-assisted interpretation across readers of varying expertise, with percentages of csPCas at each PI-RADS score and for each change in scores after the AI-assisted reading

Figure 2 illustrates the changes in PI-RADS scores between the two reading approaches and their impact on csPCa detection. Residents were most likely to change PI-RADS scores after AI-assisted readings, with 19 changes *versus* 9 for basic readers and 7 for experts. Among the 15 cases downrated from PI-RADS > 3 to lower scores (8 by residents, 5 by basic readers, and 2 by experts), 5 (33.3%) were csPCa, 60% of which were ISUP 2 tumors. Conversely, among the 8 upgrades from PI-RADS 2 to higher scores (3 by residents, 2 by basic readers, and 3 by experts), only 1 case was csPCa (12.5%).

### Diagnostic performance

**Fig. 3:**
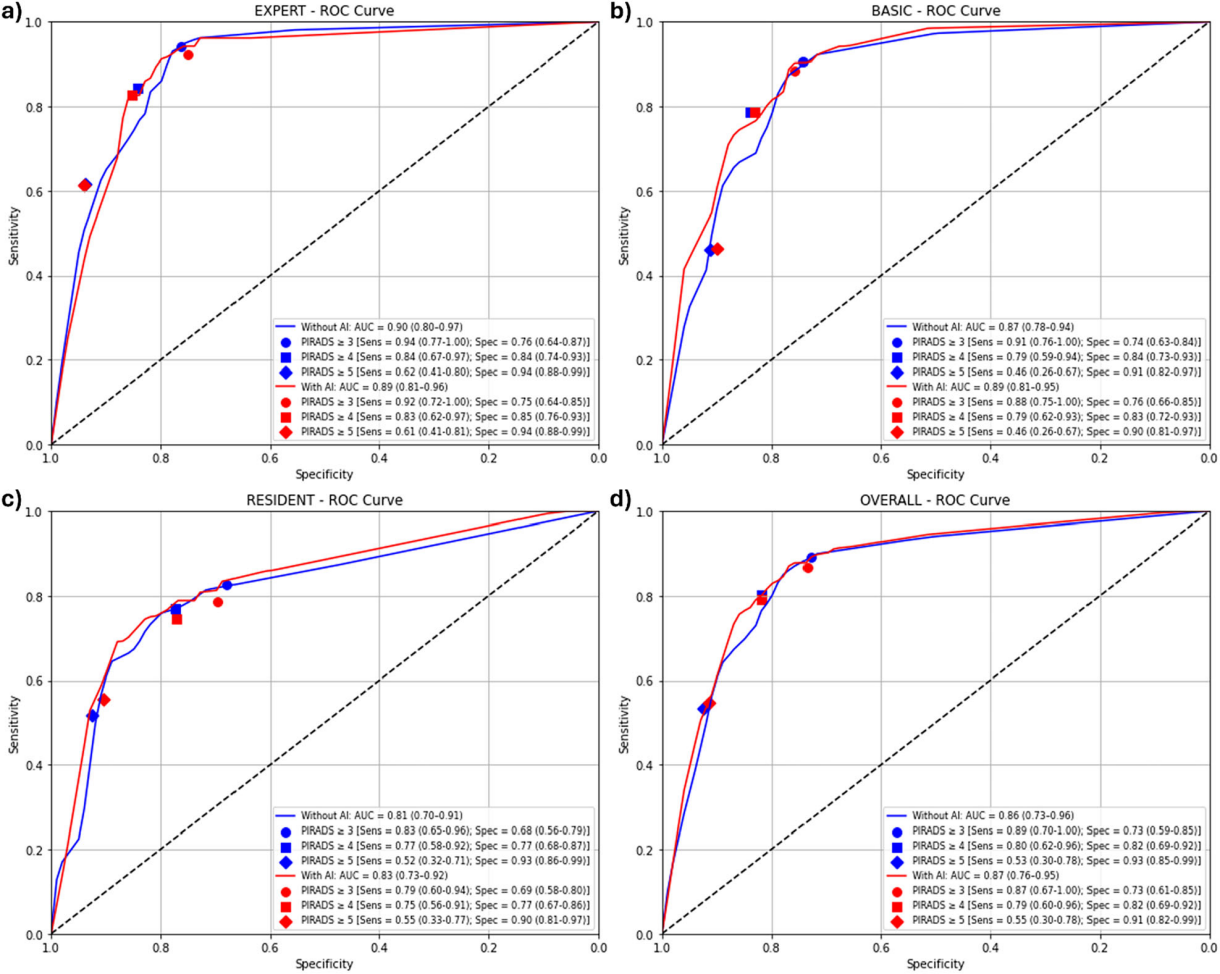
Receiver operating characteristic analysis with diagnostic performance metrics for csPCa, with and without AI assistance, stratified by reader expertise: (**a**) expert radiologists; (**b**) basic radiologists; (**c**) residents; and (**d**) overall

Figure 3, Table 3, Supplementary Fig. S2, and Supplementary Table S4 illustrate the diagnostic performance of the readings in detecting csPCa and any cancer, respectively.

**Table 3:** Patient-level detection performance for csPCa, reported for overall readings and stratified by radiologist experience level

Parameter		Radiologist only	Radiologist + AI	*p*-value
OVERALL				
AUROC		0.86 [0.73, 0.96]	0.87 [0.76, 0.95]	0.739
PI-RADS ≥ 3				
Sensitivity		0.89 [0.70, 1.00]	0.87 [0.67, 1.00]	0.739
Specificity		0.73 [0.59, 0.85]	0.73 [0.61, 0.85]	0.795
PPV		0.54 [0.35, 0.71]	0.53 [0.37, 0.70]	0.713
NPV		0.95 [0.86, 1.00]	0.94 [0.84, 1.00]	0.683
PI-RADS ≥ 3				
Sensitivity		0.80 [0.62, 0.96]	0.79 [0.60, 0.96]	0.736
Specificity		0.82 [0.69, 0.92]	0.82 [0.69, 0.92]	0.708
PPV		0.61 [0.42, 0.81]	0.61 [0.41, 0.81]	0.781
NPV		0.92 [0.84, 0.98]	0.92 [0.83, 0.99]	0.674
EXPERT				
AUROC		0.90 [0.80, 0.97]	0.89 [0.81, 0.96]	0.870
PI-RADS ≥ 3				
Sensitivity		0.94 [0.77, 1.00]	0.92 [0.72, 1.00]	0.870
Specificity		0.76 [0.64, 0.87]	0.75 [0.64, 0.85]	0.831
PPV		0.58 [0.43, 0.74]	0.57 [0.40, 0.71]	0.809
NPV		0.98 [0.90, 1.00]	0.97 [0.88, 1.00]	0.730
PI-RADS ≥ 4				
Sensitivity		0.84 [0.67, 0.97]	0.83 [0.62, 0.97]	0.871
Specificity		0.84 [0.74, 0.93]	0.85 [0.76, 0.93]	0.743
PPV		0.66 [0.48, 0.82]	0.66 [0.47, 0.84]	0.763
NPV		0.94 [0.87, 0.99]	0.93 [0.85, 0.99]	0.655
BASIC				
AUROC		0.87 [0.78, 0.94]	0.89 [0.81, 0.95]	0.707
PI-RADS ≥ 3				
Sensitivity		0.91 [0.76, 1.00]	0.88 [0.75, 1.00]	0.707
Specificity		0.74 [0.63, 0.84]	0.76 [0.66, 0.85]	0.777
PPV		0.55 [0.40, 0.70]	0.56 [0.41, 0.72]	0.695
NPV		0.96 [0.89, 1.00]	0.95 [0.89, 1.00]	0.642
PI-RADS ≥ 4				
Sensitivity		0.79 [0.59, 0.94]	0.79 [0.62, 0.93]	0.697
Specificity		0.84 [0.73, 0.93]	0.83 [0.72, 0.93]	0.660
PPV		0.63 [0.45, 0.81]	0.63 [0.44, 0.81]	0.831
NPV		0.92 [0.85, 0.98]	0.92 [0.85, 0.98]	0.690
RESIDENT				
AUROC		0.81 [0.70, 0.91]	0.83 [0.73, 0.92]	0.658
PI-RADS ≥ 3				
Sensitivity		0.83 [0.65, 0.96]	0.79 [0.60, 0.94]	0.658
Specificity		0.68 [0.56, 0.79]	0.69 [0.58, 0.80]	0.785
PPV		0.47 [0.32, 0.62]	0.48 [0.32, 0.64]	0.633
NPV		0.92 [0.83, 0.98]	0.90 [0.82, 0.98]	0.658
PI-RADS ≥ 4				
Sensitivity		0.77 [0.58, 0.92]	0.75 [0.56, 0.91]	0.654
Specificity		0.77 [0.68, 0.87]	0.77 [0.67, 0.86]	0.714
PPV		0.54 [0.38, 0.71]	0.54 [0.38, 0.70]	0.750
NPV		0.90 [0.83, 0.97]	0.90 [0.82, 0.97]	0.684

Values are estimated with 95% CIs in the brackets

*PPV* Positive predictive value, *NPV* Negative predictive value, *AUROC* Area under the receiver operating characteristic curve, *AI* Artificial intelligence

For csPCa detection, the overall AUROC of the readers did not significantly differ between AI-assisted and unassisted mpMRI assessments (*p* = 0.739), with AUROC values of 0.86 (95% CI: 0.73–0.96) without AI and 0.87 (95% CI: 0.76–0.95) with AI. At a PI-RADS cutoff of ≥ 3, AI-assisted readings demonstrated a slightly lower sensitivity of 0.87 (95% CI: 0.67–1.00) compared to 0.89 (95% CI: 0.70–1.00) without AI. Specificity remained unchanged at 0.73, with comparable positive predictive value of 0.53 *versus* 0.54 and negative predictive value of 0.94 *versus* 0.95. No statistically significant differences in performance metrics were also observed between AI-assisted and unassisted assessments across expertise groups, when using a PI-RADS cutoff ≥ 4 or for the detection of any PCa.

### Benefit- to-harm ratios

**Fig. 4:**
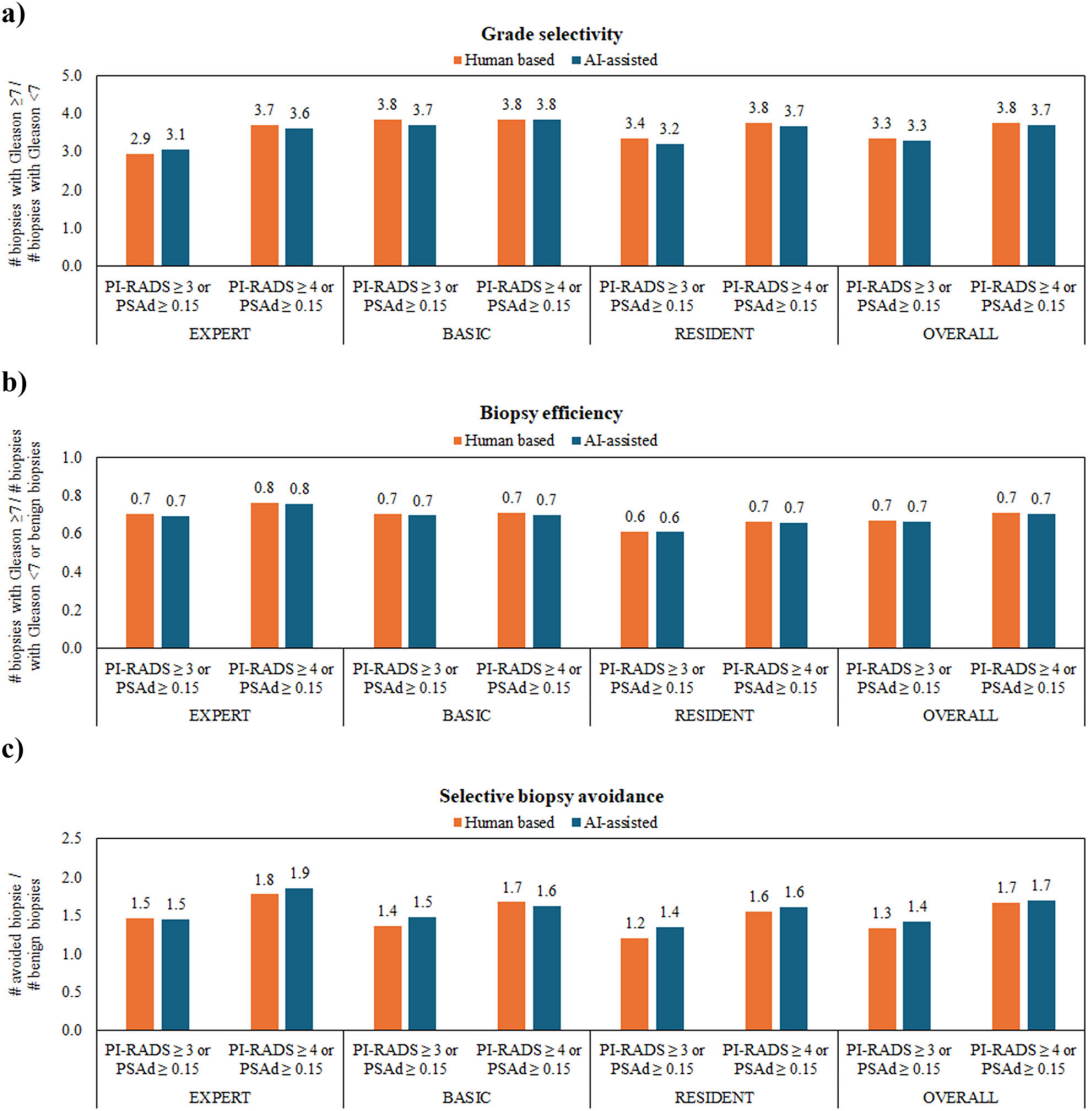
Bar plots comparing grade selectivity (**a**), biopsy efficiency (**b**), and selective biopsy avoidance (**c**) across four biopsy recommendation strategies

Supplementary Table S5 presents the proportions of Gleason ≥ 7 cancer detected, biopsies performed, Gleason < 7 cancer detected, and Gleason ≥ 7 cancer missed, overall and stratified by radiologist experience level, across the four biopsy recommendation strategies. csPCa detection rates ranged from 22.8% to 24.5%, with biopsies performed in 55.3–61.0% of cases, and fewer than 3.2% of csPCa cases were missed. Overall, the biopsy strategy based on PI-RADS ≥ 3 or PSAd ≥ 0.15 ng/mL/mL without AI assistance detected slightly more csPCa, but at the cost of a higher number of biopsies and increased detection of clinically insignificant cancers. Figure 4 shows grade selectivity, biopsy efficiency, and selective biopsy avoidance ratios across the 4 biopsy recommendation strategies, overall and stratified by radiologists’ expertise level. Raising the PI-RADS threshold from 3 to 4 improved selectivity, while AI provided a slight benefit for expert readers (3.1 *versus* 2.9) when applying a biopsy recommendation strategy based on PI-RADS ≥ 3 or PSAd ≥ 0.15 ng/mL/mL. Biopsy efficiency remained consistent across strategies, both overall (0.7) and by experience level (0.6–0.8). A PI-RADS ≥ 4 threshold improved selective biopsy avoidance (1.6–1.9) compared to PI-RADS ≥ 3 (1.2–1.5). Among residents, AI-assisted readings using a PI-RADS cutoff of 3/PSAd ≥ 0.15 ng/mL/mL resulted in a slight increase in selective biopsy avoidance (1.4 *versus* 1.2).

**Fig. 5:**
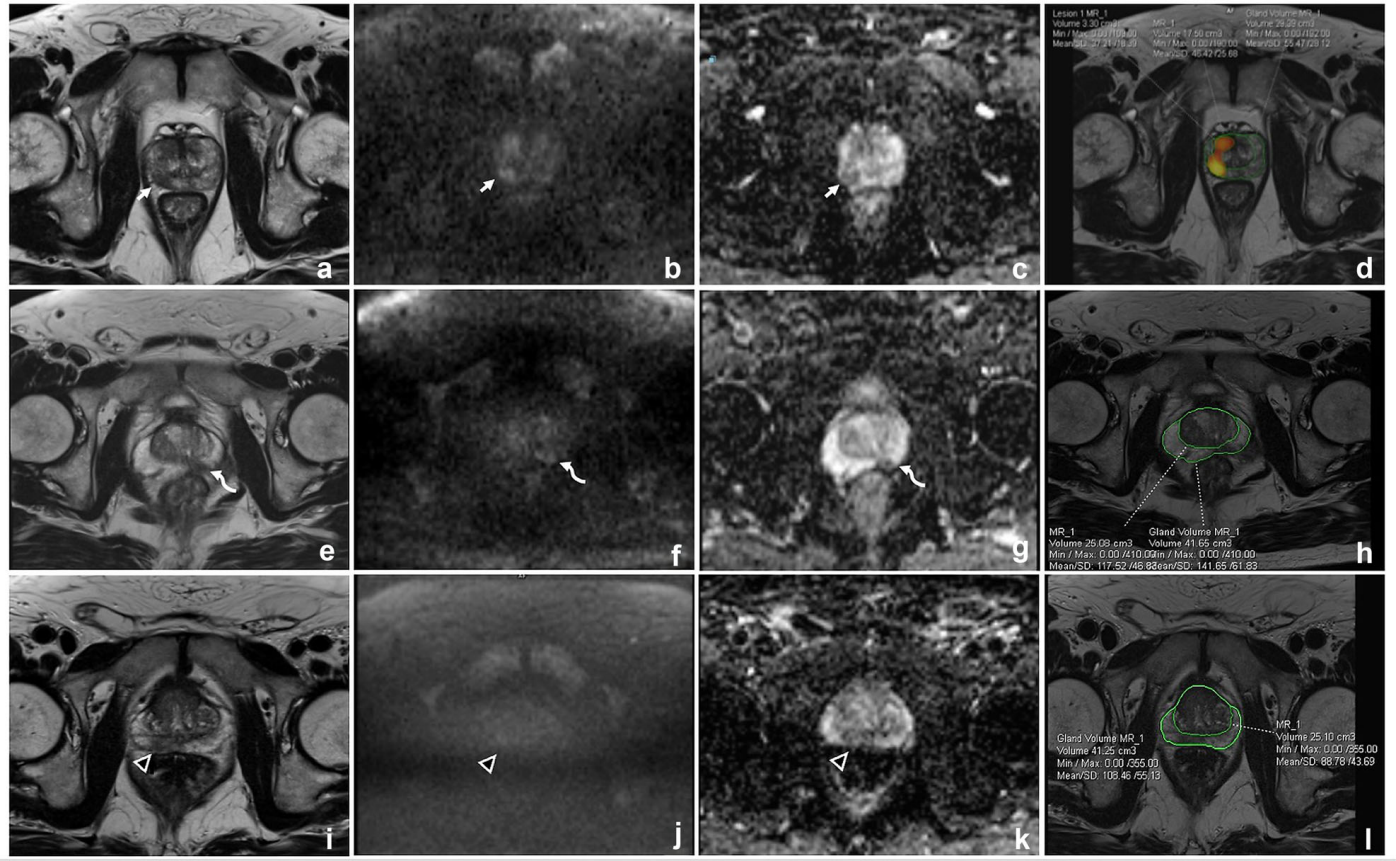
Representative examples from three patients undergoing prostate MRI for clinical suspicion of PCa. First case: Axial T2-weighted (**a**), high *b*-value DWI (**b**), and corresponding ADC map (**c**) demonstrate a lesion in the right posterolateral peripheral zone at the mid-gland (arrows), classified as PI-RADS 4 by the readers both without and with AI assistance (**d**); biopsy confirmed Grade Group (GG) 3 cancer. Second case: axial T2-weighted (**e**), high *b*-value DWI (**f**), and ADC map (**g**) show a lesion in the left posteromedian peripheral zone at the mid-gland (curved arrows), classified as PI-RADS 3 by a resident without AI assistance and downgraded to PI-RADS 2 after AI review (**h**); biopsy revealed GG 3 cancer. Third case: Axial T2-weighted (**i**), high *b*-value DWI (**j**), and ADC map (**k**) depict a lesion in the right posteromedian peripheral zone at the base (arrowheads), classified as PI-RADS 3 by the basic readers and one resident without AI assistance and downgraded to PI-RADS 2 after AI review (**l**); biopsy showed no evidence of cancer

Representative examples of changes or no-changes in PI-RADS category with AI assistance are provided in Fig. 5.

## Discussion

We evaluated a CE-marked AI system as a concurrent reader for prostate MRI across radiologists of varying experience. Intra-reader consistency for PI-RADS scores remained similar with or without AI (κ0.584 *versus* 0.573), indicating stable individual assessments. The recent data from the PI-CAI challenge reported overall PI-RADS inter-reader agreement of 73%, with higher agreement for positive cases (89%) and lower for negative cases (66%) [23]. However, the lack of improvement is contrary to prior experience that reported increased reader variability with AI detection software [24‒26]. Winkel et al, using the same AI platform that we employed, reported that inter-reader agreement improved with AI support, particularly at a PI-RADS ≥ 4 threshold, where Fleiss κ increased from 0.22 to 0.36 [24]. However, several methodological differences may explain these discrepancies: they used consensus readings of experienced radiologists as the reference standard, applied PI-RADS v2 rather than v2.1, and included a wash-out period between reading sessions [24].

Interestingly, we found that residents were more likely to assign higher overall likelihood scores when assisted by the AI, even though this assistance did not translate into a significant effect on overall PI-RADS category assignments (Fig. 2). When analyzing the impact of AI on PI-RADS scoring in detail, we observed that residents and less-experienced readers tended to modify their initial assessments more frequently after reviewing AI suggestions. These findings suggest that AI may influence less experienced readers more, likely because they are more receptive to external guidance or less confident in their initial assessments. However, the clinical impact appears limited. Of the eight cases upgraded from PI-RADS 2 after AI assistance, only one (12.5%) was confirmed as csPCa. This low yield raises concerns about a possible risk of automation bias, whereby over-reliance on AI could lead to unnecessary score upgrades and potentially unwarranted biopsies [27].

Notably, when assessing diagnostic performance, the overall and subgroup AUROC values did not differ significantly between AI-assisted and unassisted assessments (0.86 *versus* 0.87, respectively). Moreover, when applying a PI-RADS ≥ 3 threshold, key accuracy metrics remained comparable even among residents, and no significant impact was observed when using AI as a concurrent reader, particularly in terms of specificity and positive predictive value, which are the known limitations of MRI interpretations [6].

These findings contrast with those reported in previous studies [28, 29]. In particular, the retrospective analysis by Sun et al, which used a proprietary, noncommercial AI solution, found that AI assistance led to a significant improvement in diagnostic performance at the patient level, with the overall AUROC increasing from 0.86 to 0.91 [28]. This benefit was particularly marked among less-experienced radiologists, whose AUROC rose from 0.84 to 0.89. At the same time, expert readers showed no meaningful gain, suggesting that AI support may be most advantageous for those with limited experience. In the study by Labus et al, the authors demonstrated the beneficial role of assistance from the same concurrent DL software that we evaluated for less-experienced radiologists, with improvements in NPV and PPV, which increased from 0.70 to 0.81 and from 0.50 to 0.57, respectively [29]. At the same time, accuracy metrics remained largely unaffected for expert readers. This discrepancy may reflect methodological differences: the previous studies employed a two-session design with a wash-out period, whereas our study simulated real-world practice, with radiologists interpreting images first and then reviewing AI outputs before finalizing assessments. Another factor likely limiting the observed performance gains is the background of our readers. Although some had limited experience per ESUR/ESUI benchmarks, all were based at a tertiary urologic oncology center, providing greater exposure to PCa cases than in less specialized settings [19]. Additionally, readers incorporated clinical metadata, including PSAd, which is known to enhance disease detection in risk-based diagnostic pathways. These elements likely contributed to high baseline diagnostic performance, reducing the incremental benefit of AI support. An alternative workflow, such as AI-first followed by radiologist review of AI findings, may have yielded different results, as recently demonstrated by Gelikman et al [25].

Several recent studies have evaluated the standalone performance of various commercially available AI software tools [16, 30, 31]. Notably, in their retrospective study, Engel et al evaluated the standalone performance of the same commercially available AI algorithm used in our investigation, which showed significantly higher diagnostic accuracy in detecting csPCa compared to radiologist-assigned PI-RADS scores: 74% *versus* 63% for PI-RADS ≥ 4 (*p* < 0.01), and 70% *versus* 52% for PI-RADS ≥ 3 (*p* < 0.01) [30]. Of note, a prospective investigation employing the same software noted that AI assistance improved the positive predictive value for lesion detection compared with radiologists (58% *versus* 48%), at the cost of reduced sensitivity (80% *versus* 93%) for detecting csPCa [32]. In cases where radiologists had assigned PI-RADS 3 scores, the DL algorithm significantly improved per-patient specificity (21% *versus* 44%) without compromising sensitivity [32].

Uniquely, we also assessed the benefit-to-harm ratios of 4 different biopsy recommendation strategies, including incremental PI-RADS cutoff and PSAd, with and without AI assistance as recommended by Schoots et al [22]. Unsurprisingly, increasing the PI-RADS threshold from 3 to 4 consistently improved grade selectivity and biopsy selective avoidance ratios across all reader expertise levels, with minimal effect on biopsy efficiency. Using AI offered a modest incremental benefit in grade selectivity among experts when applying the PI-RADS ≥ 3 threshold combined with the PSAd strategy, potentially reducing the detection of less aggressive cancers. Furthermore, under the same biopsy strategy, AI improved selective biopsy avoidance among residents. This latter finding suggests that AI support may help standardize biopsy decision-making, reducing the number of unproductive biopsies, in settings where reader expertise is limited, and specificity is prioritized.

This study has several limitations that warrant consideration. First, its retrospective and single-center design, along with a relatively small sample size, may limit the generalizability of our findings. Future prospective, multicenter studies involving larger and more diverse patient populations are essential to extend our observations. Additionally, the absence of a wash-out period between sequential readings represents a methodological constraint, as it may have introduced recall bias. However, this approach accurately simulates real-world clinical practice, where radiologists can decide to incorporate AI outputs with clinical metadata into their assessments.

Additionally, we did not fully account for MRI-negative patients who avoided biopsies, so accuracy metrics should be interpreted relative to the evaluated cohort. However, evaluating pathway benefit-to-harm ratios can help mitigate this limitation [22]. Furthermore, we did not perform a per-lesion analysis, even though the software was trained to identify individual lesions.

In conclusion, our study found no significant gain in reader variability or diagnostic accuracy when a commercial AI was used as a concurrent reader for radiologists with varying experience levels in a simulated clinical routine. However, minimal incremental pathway benefits in the benefit-to-harm ratios were noted. These findings suggest that while AI models may perform strongly as standalone tools, their added value in assisted reading is influenced by the complex dynamics of human-AI interaction, including user trust, integration design, and potential cognitive biases. Continued research is needed to optimize how AI is integrated into MRI-driven, risk-based diagnostic pathways and to define the most effective and clinically meaningful roles for AI within radiological practice.

## Data Availability

The datasets used and/or analyzed during the current study are available from the corresponding author on reasonable request.

## Supplementary infomation

**Additional file 1: Table S1** Technical parameters of the local acquisition protocol. **Table S2**. Summary of intra- and inter-reader intraclass correlation coefficient (ICC) for patient-level likelihood of clinically significant cancer, reported for overall readings and stratified by radiologist experience. **Table S3** Summary of the Generalized Linear Mixed Model (GLMM) to estimate the effect of expertise and AI-reading on the overall likelihood score of csPCa. **Table S4** Patient-level detection performance for any prostate cancer, reported overall and stratified by radiologist experience. **Table S5**. Proportion of Gleason ≥ 7 cancer detected, biopsies performed, Gleason < 7 cancer detected, and Gleason ≥ 7 cancer missed, reported for overall readings and stratified by radiologist experience level across four biopsy recommendation strategies. **Fig. S1**. Percentage change in the patient-level likelihood of clinically significant cancer according to the experience. **Fig. S2** Receiver operating characteristic analysis with diagnostic performance metrics for any prostate cancer, with and without AI assistance, stratified by reader expertise: **(a)** expert radiologists; **(b)** basic radiologists; **(c)** residents; and **(d)** overall.

## Abbreviations

AI: Artificial intelligence
AUROC: Area under the receiver operating characteristic curve
CI: Confidence interval
csPCa: Clinically significant prostate cancer
ICC: Intraclass correlation coefficient
ISUP: International Society of Urological Pathology
mpMRI: Multiparametric MRI
MRI: Magnetic resonance imaging
PCa: Prostate cancer
PI-CAI: Prostate imaging-cancer AI
PI-RADS: Prostate imaging reporting and data system
PSA: Prostate-specific antigen
PSAd: PSA density
