# Supplementary material for: Concurrent AI-human interaction in prostate cancer MRI interpretation: More hype than help?
Source: Eur Radiol Exp. 2026 Mar 30;10:35. doi: 10.1186/s41747-026-00695-1 (PMC13035990; doi:10.1186/s41747-026-00695-1)

Concurrent AI-human interaction in prostate cancer MRI interpretation: more hype than help?

ELECTRONIC SUPPLEMENTARY MATERIAL

Table S1 Technical parameters of the local acquisition protocol

	Axial T2-weighted turbo spin-echo	Sagittal T2-weighted turbo spin-echo	Coronal T2-weighted turbo spin-echo	Axial DWI	Axial DCE
Repetition time (ms)	6,190	4,740	4,500	3,900	4,32
Echo time (ms)	150	102	102	68	1,65
Slice thickness (mm)*	3	3	3	3	3
Matrix (pixel size, mm)	288 × 384 (0.5 × 0.5)	288 × 320 (0.6 × 0.6)	288 × 320 (0.6 × 0.6)	88 × 110 (1.8 × 1.8)	157 × 224 (1 × 1)
Field of view	200 × 200	200 × 200	200 × 200	200 × 200	220 × 220

DCE Dynamic contrast-enhanced imaging, *DWI* Diffusion-weighted imaging. DWI was performed with *b*-values of 50, 500, 1,000, and 1500 s/mm^2. DCE temporal resolution was 8 s, total acquisition time was 230 s. *Without spacing.

Table S2. Summary of intra- and inter-reader intraclass correlation coefficient (ICC) for patient-level likelihood of clinically significant cancer, reported for overall readings and stratified by radiologist experience

	Radiologist ID	ICC*	*p*-value	95% CI	
				Lower limit	Upper limit
**Intra-reader (radiologist only VS radiologist+AI)**					
Expert	1	0.987	< 0.001	0.980	0.991
Expert	2	0.981	< 0.001	0.972	0.987
Basic	3	0.968	< 0.001	0.953	0.978
Basic	4	0.971	< 0.001	0.952	0.982
Resident	5	0.964	< 0.001	0.947	0.975
Resident	6	0.978	< 0.001	0.961	0.987
**Inter-reader (radiologist only)**					
Experts	1 *versus* 2	0.811	< 0.001	0.710	0.876
Basics	3 *versus* 4	0.805	< 0.001	0.724	0.865
Residents	5 *versus* 6	0.865	< 0.001	0.799	0.910
Overall	All	0.756	< 0.001	0.694	0.813
**Inter-reader (radiologist+AI)**					
Experts	1 *versus* 2	0.820	< 0.001	0.734	0.878
Basics	3 *versus* 4	0.777	< 0.001	0.686	0.845
Residents	5 *versus* 6	0.878	< 0.001	0.823	0.916
Overall	All	0.779	< 0.001	0.721	0.831

* Two-way random-effects, absolute agreement, single measure.

Table S3 Summary of the Generalized Linear Mixed Model (GLMM) to estimate the effect of expertise and AI-reading on the overall likelihood score of csPCa

Fixed effects	β	SE	*t*	*p*-value
Intercept	3.949	0.086	46.01	0.000
Reading				
No-AI	–	–	–	–
AI-reading	0.093	0.028	3.325	0.001
Expertise				
Resident	–	–	–	–
Basic	0.123	0.067	1.850	0.064
Expert	0.105	0.066	1.585	0.113
Reading: expertise				
AI-reading: basic	-0.063	0.040	-1.565	0.117
AI-reading: expert	-0.067	0.040	-1.672	0.094

Random Effects	Variance	SD		
ImageID	0.049	0.221		
RadiologistID	0.001	0.033		
Residual	0.041	0.203		

Post-hoc pairwise comparisons (no-AI *versus* AI-reading)	β	SE	z.ratio	p-value
resident	-0.093	0.028	-3.325	0.001
basic	-0.030	0.029	-1.061	0.289
expert	-0.027	0.028	-0.932	0.351

SE Standard error, *SD* Standard deviation.

Table S4 Patient-level detection performance for any prostate cancer, reported overall and stratified by radiologist experience.

Parameter		Radiologist only	Radiologist+AI	*p*-value
OVERALL				
	AUROC	0.82 [0.67, 0.97]	0.84 [0.71, 0.96]	0.692
PIRADS ≥ 3				
	Sensitivity	0.78 [0.59, 0.97]	0.76 [0.57, 0.97]	0.692
	Specificity	0.78 [0.62, 0.91]	0.79 [0.66, 0.90]	0.774
	PPV	0.69 [0.50, 0.86]	0.68 [0.50, 0.85]	0.714
	NPV	0.85 [0.72, 0.98]	0.84 [0.71, 0.98]	0.699
PIRADS ≥ 4				
	Sensitivity	0.68 [0.52, 0.85]	0.67 [0.51, 0.82]	0.694
	Specificity	0.87 [0.73, 0.97]	0.87 [0.73, 0.97]	0.710
	PPV	0.76 [0.56, 0.94]	0.76 [0.55, 0.95]	0.787
	NPV	0.82 [0.71, 0.92]	0.81 [0.70, 0.92]	0.692
EXPERT				
	AUROC	0.89 [0.77, 0.98]	0.89 [0.76, 0.98]	0.869
PIRADS ≥ 3				
	Sensitivity	0.84 [0.64, 0.98]	0.82 [0.58, 0.98]	0.869
	Specificity	0.84 [0.74, 0.93]	0.82 [0.72, 0.91]	0.806
	PPV	0.76 [0.63, 0.88]	0.73 [0.57, 0.86]	0.858
	NPV	0.90 [0.78, 0.98]	0.88 [0.74, 0.98]	0.802
PIRADS ≥ 4				
	Sensitivity	0.72 [0.55, 0.88]	0.71 [0.53, 0.87]	0.845
	Specificity	0.91 [0.80, 0.98]	0.91 [0.82, 0.98]	0.709
	PPV	0.82 [0.63, 0.97]	0.83 [0.66, 0.97]	0.778
	NPV	0.84 [0.74, 0.93]	0.84 [0.73, 0.93]	0.654
BASIC				
	AUROC	0.83 [0.72, 0.92]	0.85 [0.76, 0.93]	0.662
PIRADS ≥ 3				
	Sensitivity	0.78 [0.63, 0.91]	0.76 [0.61, 0.90]	0.662
	Specificity	0.79 [0.67, 0.91]	0.81 [0.69, 0.91]	0.725
	PPV	0.70 [0.55, 0.85]	0.71 [0.54, 0.85]	0.687
	NPV	0.85 [0.75, 0.94]	0.85 [0.74, 0.94]	0.654
PIRADS ≥ 4				
	Sensitivity	0.67 [0.52, 0.82]	0.67 [0.51, 0.82]	0.691
	Specificity	0.89 [0.77, 0.97]	0.88 [0.77, 0.97]	0.677
	PPV	0.79 [0.61, 0.94]	0.77 [0.59, 0.93]	0.857
	NPV	0.81 [0.72, 0.90]	0.81 [0.72, 0.90]	0.745
RESIDENT				
	AUROC	0.75 [0.65, 0.85]	0.79 [0.69, 0.88]	0.564
PIRADS ≥ 3				
	Sensitivity	0.72 [0.56, 0.88]	0.70 [0.55, 0.84]	0.564
	Specificity	0.71 [0.59, 0.82]	0.73 [0.62, 0.84]	0.759

PPV	0.60 [0.45, 0.74]	0.62 [0.47, 0.76]	0.621
NPV	0.81 [0.69, 0.91]	0.80 [0.68, 0.89]	0.639
PIRADS ≥ 4			
Sensitivity	0.66 [0.50, 0.80]	0.65 [0.49, 0.79]	0.572
Specificity	0.81 [0.70, 0.90]	0.81 [0.70, 0.91]	0.724
PPV	0.67 [0.51, 0.83]	0.67 [0.52, 0.82]	0.752
NPV	0.79 [0.69, 0.89]	0.79 [0.68, 0.88]	0.681

Values are estimated with 95% CI in the brackets. *AI* Artificial intelligence, *PPV* Positive predictive value, *NPV* Negative predictive value, *AUROC* Area under the receiver operating characteristic curve.

Table S5. Proportion of Gleason ≥ 7 cancer detected, biopsies performed, Gleason < 7 cancer detected, and Gleason ≥ 7 cancer missed, reported for overall readings and stratified by radiologist experience level across four biopsy recommendation strategies.

Experience level	Biopsy criterion	Gleason ≥ 7 cancer detected	Biopsies performed	Gleason < 7 cancer detected	Gleason ≥ 7 cancer missed
Overall	Radiologist only: PI-RADS ≥ 3 or PSAd ≥ 0.15	24.5%	61.0%	7.3%	1.5%
	Radiologist only: PI-RADS ≥ 4 or PSAd ≥ 0.15	23.2%	55.8%	6.2%	2.8%
	Radiologist+AI: PI-RADS ≥ 3 or PSAd ≥ 0.15	23.7%	59.3%	7.2%	2.3%
	Radiologist+AI: PI-RADS ≥ 4 or PSAd ≥ 0.15	22.8%	55.3%	6.2%	3.2%
Expert	Radiologist only: PI-RADS ≥ 3 or PSAd ≥ 0.15	25.0%	60.5%	8.5%	1.0%
	Radiologist only: PI-RADS ≥ 4 or PSAd ≥ 0.15	24.0%	55.5%	6.5%	2.0%
	Radiologist+AI: PI-RADS ≥ 3 or PSAd ≥ 0.15	24.5%	60.0%	8.0%	1.5%
	Radiologist+AI: PI-RADS ≥ 4 or PSAd ≥ 0.15	23.5%	54.5%	6.5%	2.5%
Basic	Radiologist only: PI-RADS ≥ 3 or PSAd ≥ 0.15	25.0%	60.5%	6.5%	1.0%
	Radiologist only: PI-RADS ≥ 4 or PSAd ≥ 0.15	23.0%	55.5%	6.0%	3.0%
	Radiologist+AI: PI-RADS ≥ 3 or PSAd ≥ 0.15	24.0%	58.5%	6.5%	2.0%
	Radiologist+AI: PI-RADS ≥ 4 or PSAd ≥ 0.15	23.0%	56.0%	6.0%	3.0%
Resident	Radiologist only: PI-RADS ≥ 3 or PSAd ≥ 0.15	23.5%	62.0%	7.0%	2.5%
	Radiologist only: PI-RADS ≥ 4 or PSAd ≥ 0.15	22.5%	56.5%	6.0%	3.5%
	Radiologist+AI: PI-RADS ≥ 3 or PSAd ≥ 0.15	22.5%	59.5%	7.0%	3.5%
	Radiologist+AI: PI-RADS ≥ 4 or PSAd ≥ 0.15	22.0%	55.5%	6.0%	4.0%

Supplementary information about algorithm details, including the pipeline and training

We employed a commercially available advanced imaging and visualization platform designed to support PI-RADS reporting and powered by AI-based lesion detection and classification (syngo.via MR Prostate AI, version VB50, Siemens Healthineers, Erlangen, Germany). The algorithm was trained and validated with 2,170 biparametric MRIs from seven institutions. The DLA was designed to evaluate biparametric MRI based on axial T2-weighted images (T2WIs) and diffusion-weighted images (DWIs). The system initiates with the automated segmentation of the prostate gland on axial T2-weighted (T2WI) and diffusion-weighted imaging (DWI) sequences, followed by coregistration of these sequences. From the DWI data, it generates both a synthetic high b-value image (b = 2000 s/mm²) and apparent diffusion coefficient (ADC) maps. These coregistered inputs (T2WI, synthetic *b* = 2000 image, and ADC map) are processed through a deep learning pipeline. In the first stage, a 2D convolutional neural network (CNN) identifies potential lesions, which are then further analyzed by a 3D patch-wise CNN designed to reduce false positives. Last, the PI-RADS scoring net assigns each lesion a PI-RADS category based on the AI-derived level of suspicion (LoS). PI-RADS 3 assignments are for lesions with LoS of 60-80. For lesions with LoS >80, PI-RADS 4 is assigned to lesions < 1.5 cm and PI-RADS 5 to lesions > 1.5 cm. Each of the networks for lesion candidate detection, false-positive reduction, and PI-RADS classification is trained independently. The results include a qualitative heat map indicating a maximum of 5 regions with PI-RADS category scores of 3-5, 3D lesion contours, anatomical localization, estimated PI-RADS score, and an AI-derived level of suspicion (LoS), which ranges from 60 to 100. The LoS reflects the model's confidence based on training with lesions annotated as PI-RADS ≥3 by expert radiologists. Ground truth labels were based on radiologic assessment and, when available, histopathological confirmation (e.g., biopsy or prostatectomy). Additionally, the system provides segmentation of the entire prostate, including the peripheral and non-peripheral zones and volumes. The DLA results provide PI-RADS scores and a snapshot of each of up to five radiologist-verified lesions in each patient.

Key references

- Winkel DJ, Tong A, Lou B, Kamen A et al. A Novel Deep Learning Based Computer-Aided Diagnosis System Improves the Accuracy and Efficiency of Radiologists in Reading Biparametric Magnetic Resonance Images of the Prostate: Results of a Multireader, Multicase Study. Invest Radiol. 2021 Oct 1;56(10):605-613. doi: 10.1097/RLI.0000000000000780.
- Oerther B, Engel H, Nedelcu A, et al. Prediction of upgrade to clinically significant prostate cancer in patients under active surveillance: Performance of a fully automated AI-algorithm for lesion detection and classification. Prostate. 2023 Jun;83(9):871-878. doi: 10.1002/pros.24528.
- Labus S, Altmann MM, Huisman H, et al. A concurrent, deep learning-based computer-aided detection system for prostate multiparametric MRI: a performance study involving experienced and less-experienced radiologists. Eur Radiol. 2023 Jan;33(1):64-76. doi: 10.1007/s00330-022-08978-y.
- Lee YJ, Moon HW, Choi MH, et al. MRI-based Deep Learning Algorithm for Assisting Clinically Significant Prostate Cancer Detection: A Bicenter Prospective Study. Radiology. 2025 Mar;314(3):e232788. doi: 10.1148/radiol.232788.

Fig. S1. Percentage change in the patient-level likelihood of clinically significant cancer according to the experience

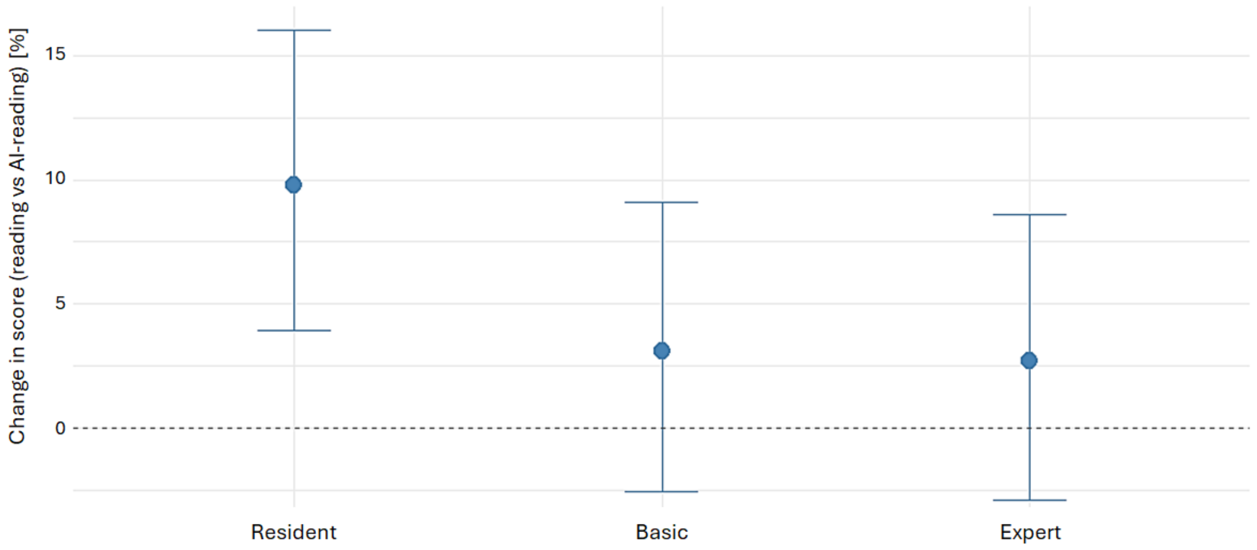

Fig. S2 Receiver operating characteristic analysis with diagnostic performance metrics for any prostate cancer, with and without AI assistance, stratified by reader expertise: (**a**) expert radiologists; (**b**) basic radiologists; (**c**) residents; and (**d**) overall.

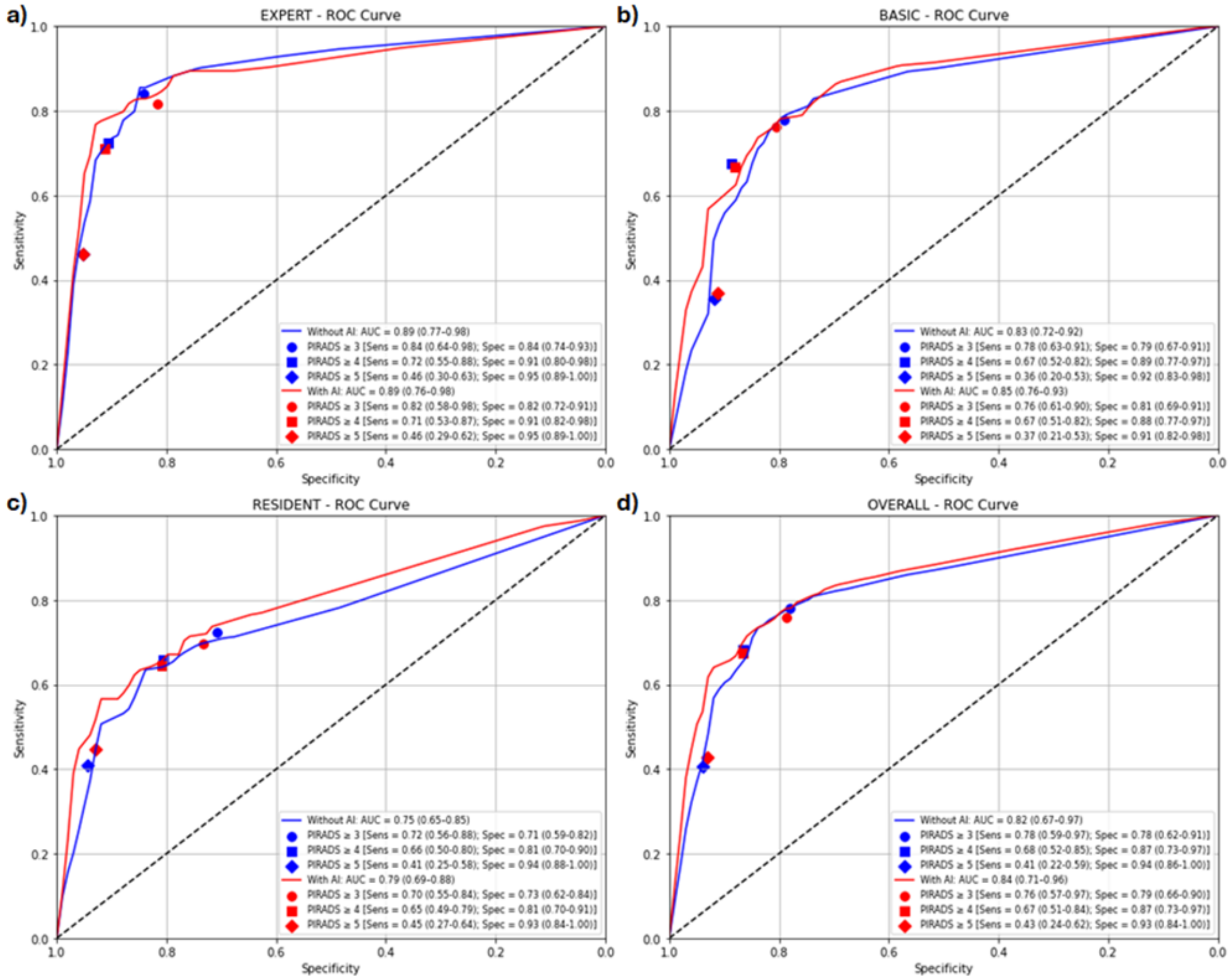

Supplement: Supplementary file 1 — Additional file 1: Table S1 Technical parameters of the local acquisition protocol. Table S2. Summary of intra- and inter-reader intraclass correlation coefficient (ICC) for patient-level likelihood of clinically significant cancer, reported for overall readings and stratified by radiologist experience. Table S3 Summary of the Generalized Linear Mixed Model (GLMM) to estimate the effect of expertise and AI-reading on the overall likelihood score of csPCa. Table S4 Patient-level detection performance for any prostate cancer, reported overall and stratified by radiologist experience. Table S5. Proportion of Gleason ≥ 7 cancer detected, biopsies performed, Gleason < 7 cancer detected, and Gleason ≥ 7 cancer missed, reported for overall readings and stratified by radiologist experience level across four biopsy recommendation strategies. Fig. S1. Percentage change in the patient-level likelihood of clinically significant cancer according to the experience. Fig. S2 Receiver operating characteristic analysis with diagnostic performance metrics for any prostate cancer, with and without AI assistance, stratified by reader expertise: (a) expert radiologists; (b) basic radiologists; (c) residents; and (d) overall. [file 41747_2026_695_MOESM1_ESM.pdf]
